# Encapsulation of Lipid-Soluble Bioactives by Nanoemulsions

**DOI:** 10.3390/molecules25173966

**Published:** 2020-08-31

**Authors:** Shahin Banasaz, Ksenia Morozova, Giovanna Ferrentino, Matteo Scampicchio

**Affiliations:** Faculty of Science and Technology, Free University of Bozen-Bolzano, Piazza Università 1, 39100 Bolzano, Italy; shahin.banasaz@natec.unibz.it (S.B.); giovanna.ferrentino@unibz.it (G.F.); matteo.scampicchio@unibz.it (M.S.)

**Keywords:** nanoemulsion, vitamin A, vitamin E, vitamin D, vitamin K, β-carotene, essential oils, encapsulation, carotenoids

## Abstract

Lipid-soluble bioactives are important nutrients in foods. However, their addition in food formulations, is often limited by limited solubility and high tendency for oxidation. Lipid-soluble bioactives, such as vitamins A, E, D and K, carotenoids, polyunsaturated fatty acids (PUFA) and essential oils are generally dispersed in water-based solutions by homogenization. Among the different homogenization technologies available, nanoemulsions are one of the most promising. Accordingly, this review aims to summarize the most recent advances in nanoemulsion technology for the encapsulation of lipid-soluble bioactives. Modern approaches for producing nanoemulsion systems will be discussed. In addition, the challenges on the encapsulation of common food ingredients, including the physical and chemical stability of the nanoemulsion systems, will be also critically examined.

## 1. Introduction

Lipid-soluble bioactives are essential nutrients that play significant role in human diet [1]. This group includes lipophilic vitamins (e.g., vitamin A, D, E and K), carotenoids, polyunsaturated fatty acids (PUFA) and essential oils. However, their direct use in food and beverage products is limited by their low water solubility and high sensitivity towards oxidation. For these reasons, these bioactives need to be encapsulated. A number of innovative technologies are fit for this purpose. Examples include coacervation, spray-drying, freeze drying, spray cooling and fluidized bed coating [2]. Alternatively, emulsification technology is also commonly used.

Emulsions generally consist of two or more immiscible liquids in a way that one of the liquids is dispersed in the other as small spherical droplets [3]. The development of droplets with a given size, structure and shape is dependent of the liquid (s), emulsification agent and the emulsification methods used. Emulsions occur usually in two categories: water-in-oil (W/O) and oil-in-water (O/W) [4]. Water-in-oil emulsions consist of water droplets dispersed in oil medium (such as butter or margarine). Instead, oil-in-water emulsions contain droplets of oil dispersed in aqueous medium, e.g., mayonnaise, beverages, milk and creams.

Emulsions can be also categorized depending on the diameter size of the droplets. Droplet size greatly influences the optical rheology, physical and chemical properties of the emulsions. Conventional or macroemulsions have a droplet size range between 100 nm and 100 µm and are thermodynamically unstable and opaque. Nanoemulsion can be defined by a smaller droplet size with a mean diameter between 20 to 100 nm and they are still categorized as thermodynamically unstable systems. Microemulsions unlike the other two are thermodynamically stable systems with a particle size between 5 nm and 50 nm [4]. Regardless to the type and size of the emulsions, the technology used for their preparation includes high or low energy methods depending on the type of equipment and power needed to produce emulsions.

Although a number of studies have investigated the possibility to improve the oxidative stability of encapsulated bioactives against environmental stress factors and increase their biological and nutritional properties [5], their high surface to volume ratio and the high oxygen diffusion in the aqueous phase may increase the lipid oxidation process [6].

To overcome such drawback, several technologies have been proposed in recent years. Among others, nanoemulsion technology seems one of the most promising, not only for delivering lipid-soluble bioactives in foods, but also for protecting them from oxidation processes [7,8]. A growing number of studies suggest that smaller particle size of the droplets containing lipid-soluble bioactive compounds increase their uptake in biological systems [9,10]. For these reasons, nowadays, many food products, such as soft drinks, butter, ice-cream, milk, dressings, sauces, and creams are produced with application of nanoemulsion technologies. Nanoemulsions with desired composition, stability and functional properties can be prepared using commercial emulsifiers, oils and water using simple operations, such as mixing and homogenization.

This review focuses on the recent encapsulation studies of lipid-soluble bioactives by nanoemulsion. This article concentrates on current advances in nanoemulsion production methods, most common ingredients, and recent applications for encapsulation of lipid-soluble vitamins, PUFAs and essential oils.

## 2. Nanoemulsion Technology

### 2.1. Preparation of Nanoemulsions

**Figure 1:**
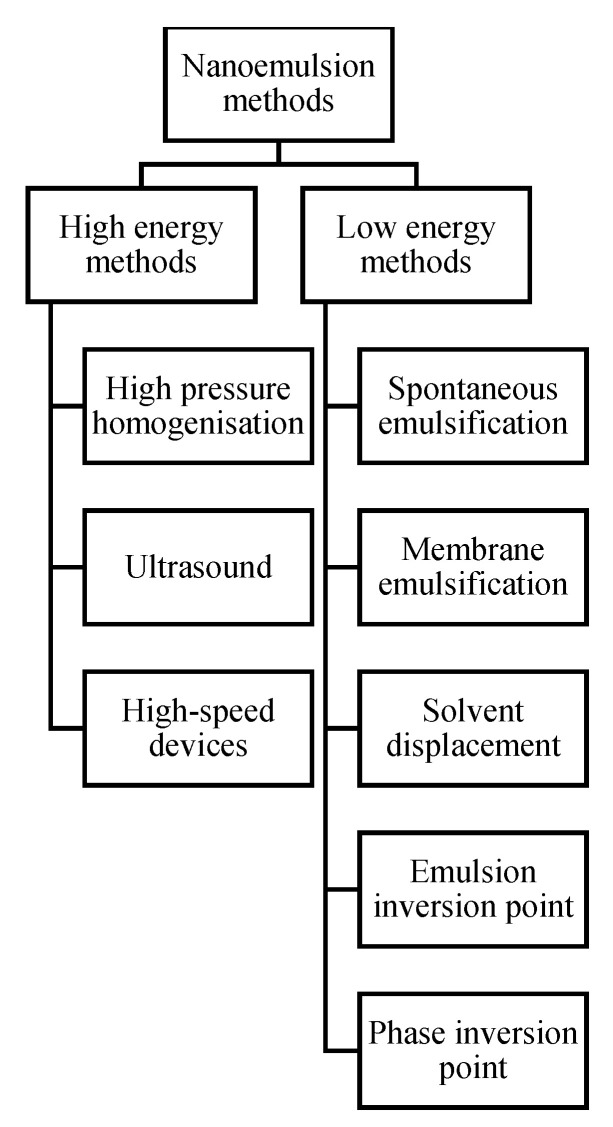
Methods for preparation of nanoemulsions.

Preparation of nanoemulsion is generally performed by using two different approaches, respectively based on high-energy or low energy needs (Figure 1). High-energy methods imply the application of special equipment, such as sonication devices and high-pressure homogenizers to generate powerful disruptive force to form small oil droplets by causing the breakup of oil and water phases [11,12,13]. The main factors that influence the size of droplets and the properties of nanoemulsions are the amount of energy applied and the choice of surfactants and other additives [14]. There are three main high energy methods classified according to the equipment used:

High-pressure homogenization: The small droplets are formed by high shear stress, while the mixture of oil and water phases is pumped through the restrictive valve of a high-pressure homogenizer [15,16].

Ultrasound: The mixture of oil and water phases with addition of surfactant is treated with high-frequency sound waves [17].

Homogenization using high-speed instruments: Rotor devices (e.g., Ultra-Turrax) provide mixing and heating of the ingredients’ mixture. However, the efficiency of this method is lower compared to the previous two, so it is often used as pretreatment before high-pressure or ultrasound emulsification [14,18].

In case of low-energy methods, the droplets in nanoemulsions are formed spontaneously as a result of phase transition, when the composition, operating conditions and temperature are favorable [19]. The most common low-energy methods are:

Spontaneous emulsification: In this approach, the aqueous phase containing water and hydrophilic surfactant is mixed with the organic solution consisting of oil, water-miscible solvent and lipophilic surfactant [20]. The spontaneous emulsification occurs when both phases are in contact and not in equilibrium state.

Membrane emulsification: According to this method the droplets are formed through a membrane into a continuous phase. This method requires less surfactant in comparison to high-energy methods. However, scale-up is often challenging because of the low flow rate of the dispersed phase through the membrane [16].

Solvent displacement: The organic phase consisting of lipophilic compounds is mixed in an aqueous phase containing surfactant. The formation of nanoemulsions occurs due to the fast diffusion of the organic phase into the aqueous phase. After that, the solvent is removed under vacuum [21,22].

Emulsion inversion point: In this approach, the composition of the system is changed at a constant temperature. The stable nanoemulsion is formed by gradual dilution with oil or water [23].

Phase inversion point. In this method, compared to the previous one the temperature of the process is varied, while the composition stays the same, in order to change the affinity of a surfactant to the oil or water phase [24,25]. A rapid cooling is applied to break up the emulsions retained at the phase inversion point [26,27,28].

### 2.2. Types of Emulsions

Nanoemulsions are typically divided into oil-in-water (O/W) nanoemulsions, which are produced by dispersing small oil particles in an aqueous phase, and water-in-oil (W/O) nanoemulsions consisting of small water particles dispersed in an oil phase [29]. In addition, by applying a two-step procedure, it is possible to obtain multiple nanoemulsions, specifically water in oil in water (W/O/W) and oil in water in oil (O/W/O) [30]. In detail, the preparation of W/O/W nanoemulsions is achieved by integrating the oil medium containing lipophilic emulsifier with the water phase in order to form the first W1/O nanoemulsions. After that this the first nanoemulsions are homogenized with an additional water phase (W2) containing hydrophilic emulsifier [31]. Among others, oil-in-water nanoemulsions are especially keen to stabilize, protect and deliver the lipophilic bioactive compounds due to their encapsulation in the oil phase [1].

### 2.3. Ingredients for Nanoemulsion Preparation

Food nanoemulsions usually contain a great variety of ingredients, which include emulsifiers, oils, gelling and thickening agents, antioxidants, preservatives, sweeteners, colorants, flavors, salts and many more. Every ingredient has specific functional, physicochemical, sensory, and nutritional properties, which can influence the final product characteristics. Quality of food emulsions depends in the first place on the containing ingredients, their interactions with each other and their physical location [3]. The main components of a nanoemulsion are water, oil, and surfactant, while their ratio and interaction influence its properties and physical and chemical stability. An overview of functional and physicochemical characteristics of the main ingredients present in food nanoemulsions is presented in the following part.

#### 2.3.1. Oils

The oil phase of nanoemulsion usually contains lipids, such as fats and oils, which are partially or completely insoluble in water and soluble in organic solvents. Lipids are also used in nanoemulsions acting as flavor, cloud and colorant additives. Edible oils for food nanoemulsions can be obtained from a number of sources, including plants, animals, fishes and nuts [32,33]. Oils and fats influence the nutritional, sensory, and physicochemical properties of nanoemulsions.

The carrier oil type may have a significant impact on the chemical stability, bioaccessibility and antioxidant properties of lipid-soluble bioactives encapsulated in nanoemulsions. For example, a recent study [34] with different types of oil phase (orange oil, tributyrin and corn oil) showed that lycopene is more susceptible to chemical degradation in the presence of unsaturated, long chain triglycerides.

#### 2.3.2. Emulsifiers

**Figure 2:**
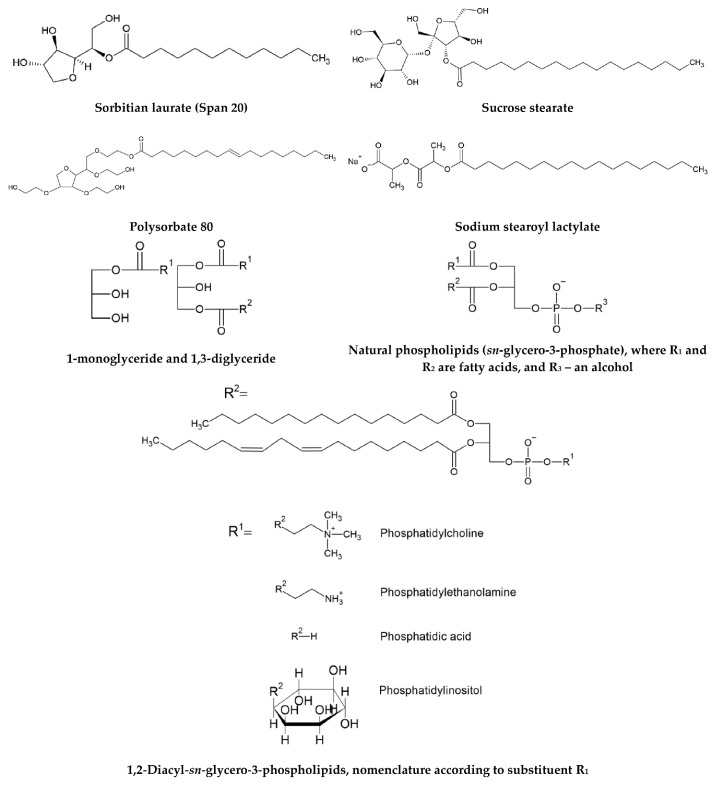
Chemical structure of some food emulsifiers.

Nanoemulsions are thermodynamically unstable systems, so that addition of chemical substances, known as surfactants or emulsifiers, is needed prior to emulsification process to form nanoemulsions that are kinetically stable for a sufficient amount of time [6,35]. Emulsifiers are surface active molecules that adsorb the oil-water interface of nanoemulsion droplets and reduce the interfacial tension, which in turn decreases the droplet size in the resulting nanoemulsion [1]. The surfactant molecules are oriented so that their non-polar parts protrude into the oil phase while the polar parts protrude into water [36]. Depending on the processing conditions and emulsion composition, different types of food grade emulsifiers can be used in the nanoemulsion composition. Some examples of food emulsifiers are shown in Figure 2. Emulsifiers define the interfacial properties of nanoemulsions: thickness, electric charge, hydrophobicity and chemical reactivity. Consequently, in order to prepare nanoemulsions for specific food applications, it is essential to choose the suitable emulsifier. Food grade emulsifiers include low-molecular-weight surfactants (natural and synthetic) and high-molecular-weight emulsifiers (mainly proteins and polysaccharides). Nowadays, there is an increasing interest to apply as surfactants saponins extracted from natural materials as surfactants [37]. Table 1 summarizes the main surfactants used for nanoemulsion preparation. In many studies for encapsulation of lipid-soluble bioactives in nanoemulsions a combination of oil-soluble and water-soluble emulsifiers is used to create stable nanoemulsions [38,39,40].

#### 2.3.3. Other Ingredients

Beside these ingredients, there are some additional agents that might be used for nanoemulsion preparation:

Weighting agents are applied in the formation O/W nanoemulsions to increase the density of triglyceride and flavor oils to the level of the aqueous phase in order to reduce the tendency for gravitational separation and creaming. Weighting agents include ester gum, brominated vegetable oil, sucrose acetate isobutyrate, rosin gum [42].

Texture modifiers are substances that are usually included in the continuous phase of emulsions to achieve modified rheological properties, including gelling agents and thickening agents. Thickening agents typically comprise of soluble polymers with an extended structure, which can achieve higher solution viscosity since it can modify the fluid flow profile. Gelling agents create chemical or physical cross-linking with other molecules and give solid-like properties to a nanoemulsion solution. The addition of texture modifiers can inhibit the droplet movement and retard gravitational separation of the nanoemulsion [29].

### 2.4. Techniques for Measuring Physical and Chemical Stability of Nanoemulsion

#### 2.4.1. Physical Stability

After formation of nanoemulsions, several physical properties can be studied such as the droplet size, the stability, crystallinity or morphology. These properties are important and are applied because nanoemulsions may be subjected to some phase separation mechanisms such as flocculation, coalescence, sedimentation and Ostwald ripening [36]. Therefore, it has been shown that some techniques can be suitable for the characterization of nanoemulsions physical stability.

Dynamic light scattering (DLS) is used for the determination of particle size distribution in solutions or suspensions. DLS measures the particles’ Brownian motion, which is related to the particle size through the Stokes-Einstein equation:

(1) $$D\;=\frac{kT}{6\pi \eta R}$$

where D is the coefficient of translation diffusion, R is the radius of the particles, k is the Boltzman’s constant, T is the absolute temperature and η is the viscosity of the medium. The droplets or particles are illuminated by a laser and through the measurement of the intensity of the scattered light, DLS allows the determination of the particles size [43,44,45].

**Figure 3:**
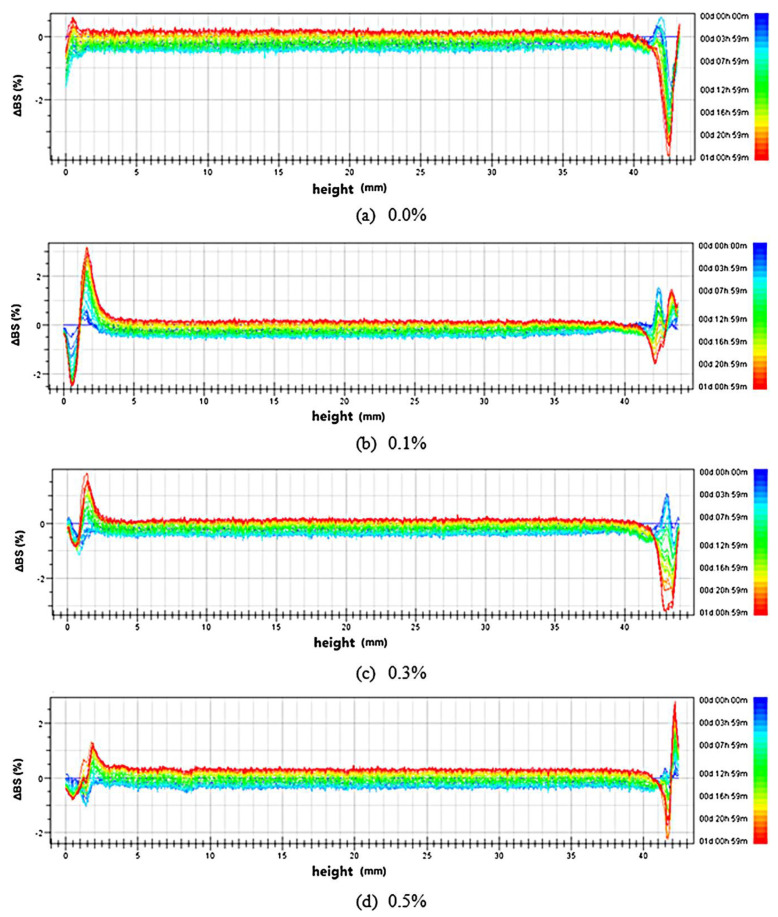
Effect of lycopene content (**a**—0.0%, **b**—0.1%, **c**—0.3%, and **d**—0.5%) on the stability of nanoemulsions measured by Turbiscan^®^ 24 h. The lines of different colors represented the changes in the light back scattering over time [47].

Physical stability and average particle size of nanoemulsion can be also addressed by multiple light scattering. The technique follows the principle that droplets in a suspension are able to scatter the radiation based on their particles size, shape and composition [46]. In most of the published studies, measurements are performed with an optical analyzer called Turbiscan^®^ (Formulation, L’Union, France). The system is composed of stations where cylindrical glass cells containing nanoemulsion can be loaded. The detection head consists of a pulsed near-infrared light source (λ = 880 nm) and two synchronous detectors for transmission and back scattering. The transmission detector catches the light passing through the sample (at 180° from the incident beam), while the back scattering (BS) detector receives the light scattered backwards by the sample (at 45° from the incident beam). The principle of the measurement is based on the particle migration and variation on particle size, resulting in discrepancy of transmission and back scattering signals. In more detail, back scattering is linked to the particle mean diameter and particle volume fraction through the following equations:

(2) BS=1(λ*)1/2

where:

(3) λ*(d,ϕ)=2d[3ϕ(1−g)Qs]

and λ* (μm) is the photon transport length, d (μm) is the particle mean diameter, ϕ (%) is the particle volume fraction, g and Qs, are two optical parameters derived from the Lorenz–Mie theory. Concerning T values, they are derived from the Lambert-Beer law, which relates the quenching of light to the concentration of the material through which the light is passing, as follows:

(4) $$T\left(\lambda ,\;r_{i}\right)=T_{0\;}e^{-3r_{i}\phi Qs/d}$$

where T_0_ is transmission value at time zero and r_i_ is the internal radius of the cylindrical vials in which the sample is loaded for the measurement. The technique was applied to monitor the physical stability over time of oil-in-water nanoemulsions stabilized by OSA starch for the encapsulation of lycopene [47]. The back scattering signals are reported in Figure 3. They were almost constant at the 3–41 mm sample height within 24 h, regardless of the lycopene concentration. However, in the bottom (0–3 mm) and the top (41–43 mm) layers, the values changed with time showing significant changes after 24 h (red lines). This indicated a physical instability of the nanoemulsions due to an increase in the droplet diameter induced by coalescence or Ostwald ripening phenomena.

Zeta Potential is a technique that measures the electro-kinetic difference of potentials between the stationary layer of fluid surrounding the droplets and the dispersion medium. A measurement of 30 mV (negative or positive) is considered as arbitrary value that splits low-charged surfaces from highly charged surfaces. Zeta potential measurement can be linked to the colloidal stability of dispersions and nanoemulsions. Its magnitude indicates the degree of electrostatic repulsion between adjacent, similarly charged particles in dispersion. High zeta potential values indicate that the systems are comprised of molecules and particles that are small enough to be physically stable without addressing aggregation phenomena. On the other hand, low zeta potential values indicate that attractive forces may exceed the electrostatic repulsion and the dispersion may encounter physical instability with breaking and flocculation phenomena. Therefore, dispersions with zeta potential values from 0 to ±30 mV suggest instability, whereas values higher than ±30 mV are typical for stable systems.

Differential Scanning Calorimetry (DSC) may be also used to detect the phase transitions, i.e., the melting of crystalline regions, and quantify the amount of fat droplets in nanoemulsions [48]. Thanasukarn et al. (2004) collogues demonstrated that the crystallization of fats influences the emulsion stability depending on the type of emulsifier used and the thermal degradation of the encapsulated bioactive. Strasdat and Bunjes used this technique to characterize lipid nanoparticle dispersion into calcium alginate beads [49]. They observed that the lipid nanoparticles with the smallest size led to a melting behavior characterized by the presence of multiple melting peaks, which allowed the characterization of the lipid particles.

Microscopy may be also used as a method to analyze nanoemulsions. The technique allows one to obtain details about the size, shape, and aggregation state of droplets inside nanoemulsions. One of the imaging methods applied for the characterization of nanoemulsions is Transmission Electron Microscopy (TEM). This method is broadly used in material and biological science. The samples should be very thin and able to resist the high vacuum present inside the instrument. Bouchemal et al. [20] investigated the use of TEM combined with bright field imaging at increasing magnification and diffraction modes to analyze the size and form of the oil-in-water nanoemulsions. They successfully examined the crystalline or amorphous character of the components inside the nanoemulsions. Yaun et al. [50] and Chu et al. (2007) [21] investigated the microstructure and the particle-size distribution of nanoemulsions demonstrating that β-carotene particles showed spherical morphology with a mean diameter of 20 nm confirming the findings obtained by DLS.

**Figure 4:**
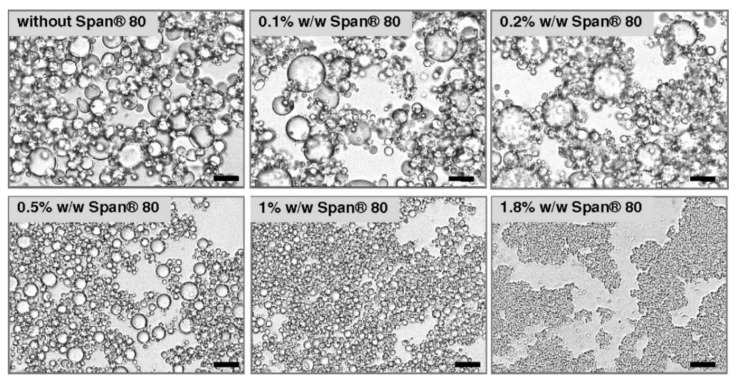
Optical microscopy images of W/O emulsions containing a paraffin oil and 1.8% (*w*/*w*) of silica particles and different concentrations of Span 80 surfactant [52].

Scanning Electron Microscopy (SEM) is also an imaging technique able to produce high-resolution three-dimensional images, which can be used to evaluate the surface structure of nanoemulsions. In general, SEM resolution is lower compared to TEM. However, SEM has a much greater depth of field, is capable to analyze bulky samples, and can better illustrate the 3D structure of the sample. The combination of larger depth of field, higher magnification, greater resolution, and relative simplicity of sample preparation make SEM one of the widely used microscopy technique for nanoemulsions analysis giving the topography of the sample surface [51]. In a study done by Nesterenko and colleagues water-in-oil emulsions were stabilized by a dual emulsifier system, including hydrophobic silica particles and Span 80 surfactant [52]. The authors studied the effect of the increasing addition of Span 80 on the emulsion morphology and stability by optical microscope. The optical microscopy images (Figure 4) showed that the droplet size of the emulsion depends on the Span 80 concentrations. For increasing concentrations of the surfactant (from 0 up to 1.8% *w*/*w*), the droplet size decreased. This effect was associated with the lower water/oil interfacial tension, which reduces the free energy required to create new interfaces and acts on the emulsion stability.

Atomic Force Microscopy (AFM) is also applied to characterize nanoemulsions. The high resolution (± 0.1 nm) achieved using AFM allows the direct viewing of single molecules or atoms. AFM depends on the raster scanning of a nanometer-sized sharp probe over a sample that has been trapped onto a carefully selected surface (glass or mica). The obtained result is a high-resolution three-dimensional profile of the surface under study. AFM can be applied on any type of material surface and images obtained by AFM are complementary to other established techniques. It has been applied for the structural characterization of, polysaccharides, proteins and liposomes. Preetz et al. [44] and Zhang and Zhao [53] demonstrated the possibility to detect differences between nanocapsules and nanoemulsions by studying the shape and morphology properties through AFM.

#### 2.4.2. Chemical Stability

Nanoemulsions are characterized by low droplet size, which makes them highly susceptible of chemical instability due to the droplets dimension due to high oil-water contact area. Lipid-soluble bioactive compounds, such as carotenoids or vitamins, can be exposed more easily to reactive substances present in the surrounding aqueous phase and can be easily destroyed by heat, light, oxygen and free radicals. Studies published on the degradation of such compounds used spectrophotometric methods or liquid chromatography to control and detect their chemical instability. UV-VIS spectroscopy was used to monitor chemical stability of vitamin D [54] β-carotene [55], and vitamin E [56].

Liquid chromatography is another common method to assess the chemical stability of lipid-soluble bioactives encapsulated in nanoemulsions, such as β-carotene [57], lycopene [58], vitamin E [59] and vitamin A [60].

The fourier transform infrared-based technique (FTIR) is an analytical technique able to measure the quantity of components in a mixture and determine the quality of a sample. It is a very sensitive method, which make measurements reproducible and accurate. Araújo et al. [43] used this technique to detect the crystallization of encapsulated thalidomide in nanoemulsions. They observed that, regardless of the polymorph employed (α- or β-), drug crystallization occurred in α- form. Zhang and Zhao [53] used FTIR spectrum to examine the tea polyphenol-Zn complex interaction mechanisms with β-chitosan based nanoparticle structures. They observed the well-incorporated characteristic peaks of tea polyphenol and tea polyphenol-Zn complex in β-chitosan nanoparticles. In the work of Campani et al. [61], FTIR was used to monitor vitamin K stability. For some lipid-soluble bioactives, such as essential oils and omega-3 fatty acids, peroxide value and fatty acid analysis with gas chromatography are often used to measure the chemical stability [62,63,64].

## 3. Encapsulation of Lipid-Soluble Bioactive Compounds by Nanoemulsions

**Figure 5:**
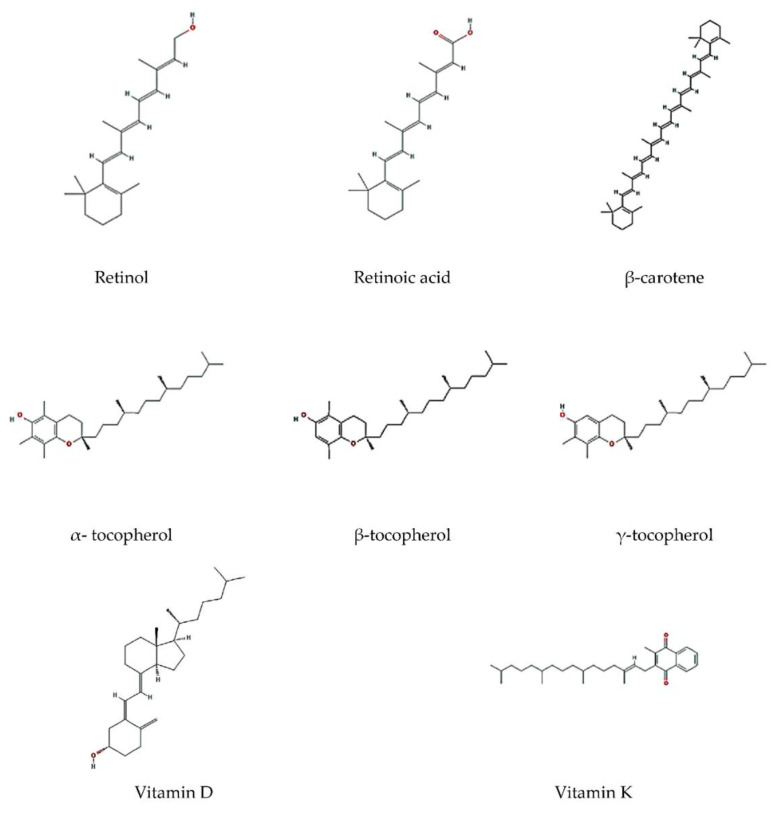
Chemical structures of some lipid-soluble vitamins.

Lipid-soluble vitamins include vitamins A, D, E, carotenoids (including β-carotene) and vitamin K (Figure 5). These vitamins have important functional properties for human body, although they also have low water solubility and are chemically instable. These properties limit their application in food industry.

Vitamin A plays an important role in ocular function, bone development and participates in strengthening the immune system [65]. Vitamin D is essential for bone metabolism and has demonstrated anti-inflammatory and immune-modulating properties [66]. Vitamin E and carotenoids belong to the most active biological antioxidants. Vitamin K, instead, is involved in calcium metabolism, bone formation and has a positive effect on cardiovascular system [67].

Nanoemulsions are an excellent option for encapsulation of lipid-soluble bioactives as they, on one hand contain lipids, in which the bioactives can be dissolved, and on the other are relatively transparent and can be incorporated into various food products and beverages. Moreover, the nanoemulsions can be further spray-dried or freeze-dried in order to obtain different solid formulations [1]. In this part, the most recent advances in encapsulation of liposoluble bioactives by nanoemulsion technology are presented.

Applications for encapsulation of lipid-soluble vitamins and some other bioactives are summarized in the Table 2. Most of them apply oil-in-water nanoemulsion systems prepared by high-energy or low-energy methods. Main ingredients (emulsifiers, oil and aqueous phase composition) and information about the particle size of the droplets are included in the table.

### 3.1. Vitamin A

The encapsulation of vitamin A in nanoemulsions is challenging because of its low water solubility and high sensitivity to oxidation. Only a few studies have been reported. Hwang and colleagues prepared a phospholipid-based microemulsion system to overcome the solubility problem of all—trans retinoic acid. Their microemulsion was prepared by mixing soybean oil and phospholipids with a high-pressure homogenizer. Eight cycles at 150 MPa were needed. However, the resulting stability was still limited. In 1 h, 9% of all-trans-retinoic acid was degraded. After 7 h, around 59% of all trans retinoic acid was retained [85].

In 2012, O/W nanoemulsions containing retinyl acetate were prepared using an ultra-high-pressure homogenizer. The retinyl acetate was dissolved in ethanol in dark conditions and consequently added to the oil phase (peanut oil). The coarse emulsion was prepared by mixing oil phase and the aqueous phase containing whey protein. The coarse emulsion was then processed two times in a ultra-high-pressure homogenizer at 200 MPa. The resulting nanoemulsions (droplet size < 300 nm) were used for preparation of retinyl acetate micelles for in vitro evaluation of their biological functions [89]. Tanglao et al. (2019) also used whey proteins (polymerized) to encapsulate vitamin A in virgin coconut O/W emulsions. Retinyl acetate was dissolved in virgin coconut oil under continuous stirring. The final emulsions were prepared by slow addition of the oil phase to the aqueous phase using a hand mixer at three different speeds. The results obtained by differential scanning calorimetry and microscopical analysis showed that encapsulation in emulsion improved thermal stability of the vitamin A [90].

The capacity of multiple nanoemulsion systems to encapsulate and protect vitamin A from oxidation has been also investigated. Yoshida and colleagues in 1999 studied vitamin A stability in oil in water (O/W), water in oil (W/O), and oil in water in oil (O/W/O). O/W/O emulsions were prepared by a two-step emulsification method [53]. First, vitamin A was dissolved in liquid paraffin and then the mixture was emulsified in 1,3-butanediol. Then, such O/W emulsion was further emulsified in oil gel that contained an organophilic clay mineral, nonionic lipophilic surfactants and liquid paraffin. The resulting stability of retinol in the O/W/O emulsion was much higher than the other types of emulsions. Shelf-life studies at 50°C showed that the amount of vitamin A retained in the O/W/O system was about 57%, much higher than in W/O (46%), and O/W (32%) systems. The reason for the better stability in O/W/O systems is probably due to a higher barrier to oxygen diffusion [60].

Many studies show that vitamin A enhances its bioavailability when formulated in nanoemulsions. For example, Kim et al. (2019) prepared vitamin A nanoemulsions to enhance the synthesis of milk-specific proteins in bovine mammary epithelial cells. Nanoemulsions were prepared by high-pressure homogenization by adding lecithin as an emulsifier. About 10 wt. % of vitamin A was mixed with ethanol (10 wt. %), soybean lecithin (5 wt. %) and 75 wt. % of water. The emulsion was mixed with a mixing homogenizer for 4 min at 24,000 rpm to obtain the coarse emulsion. Then, the final emulsion was prepared by an Ultra-Turrax blender. After cooling, the emulsion was passed through a high-pressure homogenizer at 1000 psi for three cycles. The particle size and polydispersity and zeta potential of the emulsified vitamin A were measured by dynamic light scattering method. The results showed that oil phase can increase the efficiency, stability, particle size and zeta potential of the vitamin A. Moreover, the addition of emulsified vitamins to bovine mammary epithelial cells were more effective to promote milk-specific protein synthesis in comparison to free vitamins [87].

Choudhry et al. and colleagues investigated the protective effect of saponin nanoemulsions containing vitamin A and E (10%) against oxidative stress caused by reactive oxygen species in cells. In this study, nanoemulsions with saponin (1%) were prepared by high-pressure homogenization at 25 kpsi for seven cycles. The authors demonstrated that vitamins were more effective to suppress lipid peroxidation, protein carbonylation, and DNA damage reactions in emulsified state compared to their non-emulsified form. In another recent study, nanoemulsions containing Tween 80 in combination with various encapsulating agents (Capsul, maltodextrin, sodium caseinate) were successfully used to encapsulate vitamins A and E for application in extruded feed products [89].

### 3.2. Vitamin E

Vitamin E defines a group of liposoluble vitamins widely used as antioxidants in food, pharmaceutical, and cosmetic formulations [101]. Various structural forms of vitamin E can be classified as tocopherols derivatives (α, β, γ, and δ) or as tocotrienols (α, β, γ, and δ). The α-tocopherol is considered the strongest antioxidant form among tocopherols. Although vitamin E is one of the most important oil-soluble antioxidants, hydrophobic properties limit its direct dispersion in beverages and food products containing high amounts of water. In addition, vitamin E easily degrades when exposed to oxygen, heat and light. Thus, its encapsulation in nanoemulsions facilitates its incorporation in food products [59].

There are several recent studies focusing on α-tocopherol encapsulation using emulsions with droplet diameter d in the range of d < 100 nm to d > 200 nm. In 2016 Dasgupta and colleagues studied fabrication of vitamin E nanoemulsions prepared by low energy approaches. In this study nanoemulsion was prepared using washed out method: Tween 80 (5.0% *w*/*w*) as surfactant was mixed with vitamin E acetate (2.0% *w*/*w*), and the mixture was dissolved in the mustard oil phase (3.0% *w*/*w*). The vitamin E was completely dissolved in oil phase. The aqueous phase was preheated at 75°C and slowly added to the oil phase with constant stirring. The mixture centrifuged in 25°C and at 400 rpm for 15 min. The result indicated the nanoemulsion was stable at room temperature for 15 days and the average particle diameter was 86.45 ± 3.61 nm [102].

Raikos et al. showed that vitamin E stability can be greatly improved by encapsulation in nanoemulsion system. The authors studied the effects of different thermal conditions (63 °C for 30 min, 80 °C and 90 °C for 45 s) on the stability of orange oil (3.5% *w*/*w*) beverage emulsions containing vitamin E during shelf-life study at 4 °C for four weeks. Heating process had a positive effect on colloidal stability of emulsions, which was measured by the Turbiscan. The results indicated that heating caused a mild conformational change at the whey proteins structure which subsequently had an influence on emulsion stability. Beverages samples which induced to high temperature processing for shorter time had the highest colloidal stability during 28 days of storage at 4 °C. The study concluded the retention amount of vitamin E (85%) was satisfactory for all beverage samples under the specified storage time and processing conditions, which indicates the fact that emulsions had great delivery systems potential for encapsulation of lipophilic bioactive compounds [92].

In 2013, a method for incorporation of vitamin E O/W nanoemulsions into the functional food and beverage products was studied using medium chained triglycerides as an oil phase (10%) and two surfactants. Tween 80 or Q-Naturale as surfactant was dissolved into phosphate buffer solution as an aqueous phase. A coarse emulsion was prepared by mixing the lipid and aqueous phases together by an Ultra-Turrax homogenizer. In this study, the particle mean diameter of nanoemulsions were measured by using light scattering techniques. The result of this study indicated that Tween 80 was more effective for production of nanoemulsion resulting in small droplets in lower vitamin loadings (40%), while Q-Naturale surfactant was more effective at in the higher emulsion vitamin loadings (60% to 80%). The particle size of the emulsion droplets could be decreased by reducing the concentration of the vitamin inside the oil phase [103].

In 2019, the effect of two plant-based (arabic gum and Quillaja saponin) and one animal based (whey protein isolate) surfactants was studied on the stability of fortified emulsions with vitamin E. In this study water phase was prepared by adding (1.5% *w*/*w*) of emulsifier in to the phosphate buffer at pH 7.0. Oil phase consisted of 20% (*w*/*w*) of vitamin E and 80% of corn oil. Coarse emulsions were prepared by passing 10% of the oil phase and 90% *w*/*w* of water phase through a microfluidizer for three cycles at 12,000 psi. The stability impact of the three mentioned surfactants on the emulsions was studied while keeping at room temperature at pH 7.0. The mean particle size of the nanoemulsions prepared with Quillaja saponin and whey protein isolate did not change significantly during 28 days of storage. Moreover, no creaming or flocculation phenomena was observed by both microscopy and visual inspection analysis. This was due to relatively small particle dimensions of the oil droplets inside the emulsion samples, which made them more stable to colloidal unstable phenomena such as aggregation and creaming. For both emulsions ζ-potential remained constant and highly negative during the storage time [59].

In another study from 2015, vitamin E acetate nanoemulsions were prepared with whey protein isolate and arabic gum using different ratios of orange oil and vitamin E-acetate (between 0 and 100% vitamin). Aqueous phase contained whey protein isolate and arabic gum and 10 mM sodium phosphate as buffer solution at pH 7.0. For oil-in-water nanoemulsions preparation, 10% (*w*/*w*) lipid phase (orange oil and vitamin E-acetate) was mixed with 90% (*w*/*w*) of aqueous phase and homogenized by a two-step process. First coarse emulsion was prepared by mixing lipid and aqueous phase together by an Ultra-Turrax blender. The results revealed differences between the potential ability of natural emulsifiers to stabilize nanoemulsions delivery systems as function of environmental conditions. The best results were achieved with gum arabic to protect the bioactive against environmental stress, but on another hand it was the least effective emulsifier at forming the small droplets. The nanoemulsions produced by adding gum arabic were stable to a wide range of factors such as: pH values (2–8) and ionic strengths (0–500 mM), and temperatures (30–90 °C). Nanoemulsions prepared with whey protein isolate were the most sensitive to environmental conditions changes and exhibited colloidal instability at pH value of pH 5 and elevated salt concentrations of higher than 100 mM and temperatures higher than 60°C [70].

Saberi et al. studied the influence of the oil phase and surfactants on the mean particle size of vitamin E nanoemulsions prepared by a low-energy method of spontaneous emulsification. Spontaneous emulsification was achieved by adding an organic phase to an aqueous phase at the pH 3.0, with 0.8% citric acid and 0.08% sodium benzoate. For emulsion preparation, the 10% of oil phase, 10% of surfactant were mixed with 80% of aqueous phase with a magnetic stirrer at 500 rpm at room temperature. The results indicated oil composition had a significant impact on the mean particle size of the droplets. The best results were achieved with a minimum 8% of vitamin E with addition of 2% medium chain triglycerides. Among different non-ionic surfactants studied in this research (Tween 20, 40, 60, 80, and 85), Tween 80 could better stabilize nanoemulsion samples and produce the smallest mean particle size. The results indicated that mean particle size varied by changing the concentration of Tween 80. Mean droplet size could be decreased by increasing the temperature and the speed of stirring [93].

The nanoemulsion production conditions are also important for high-energy methods. In a study performed in 2011, the formation of vitamin E-enriched oil-in-water (O/W) nanoemulsion with low fat using Tween 40 nonionic surfactant was studied by using a of high-pressure homogenization. The effect of different process conditions on nanoemulsion was measured: pressure, temperature, and content of the emulsifying ingredient. The effect of pressure on the particle size was measured during different homogenization process steps at 60 °C. The results clearly indicated an inverse correlation between the pressure and mean droplet diameter size. Droplet formation in the range between 200 and 500 nm was obtained by applying the pressures of 400 and 500 bar at the first homogenization stage, while 200 bar pressure produced droplets around 400 nm after three cycles of high-pressure homogenization. Moreover, there was a linear correlation observed between pressure and mean droplet diameter in the range of 200 to 500 bar at 60 °C. Similar results were obtained by comparing low fat (1%) and high fat content (10%) emulsions regarding the particle size distribution and mean droplet diameter [94].

### 3.3. Vitamin K

Vitamin K includes a family of lipophilic compounds with a same chemical structure containing 2-methyl-1,4-napthoquinone. Vitamin K1 (2-methyl-3-phytyl-1,4-naphthoquinone; VK1) is a molecule, which exists in a numerous number of green species of vegetables and was shown to prevent skin diseases.

Currently the number of studies on encapsulation of vitamin K by nanoemulsion technology is still limited. A study performed by Campani and colleagues aimed to solve some problems related to semisolid vitamin K1 for incorporation into aqueous lipid free formulation. In this study the nanoemulsions were prepared by using spontaneous emulsification method using Tween 80 as a surfactant and ethanol as organic solvent. The oil phase consisted of α-tocopherol and vitamin K. The organic phase was slowly mixed by a syringe pump at the flow rate 50 mL/min into an aqueous phase, while stirring at 700 rpm in the beginning and at 1400 rpm for the last 5 min. The prepared nanoemulsion was found to be a good option for commercial development of tropical vitamin K1 delivery in both liquid and aqueous formulations [61].

A research work done in 2017 studied the antitumor effect of vitamin K2 nanoemulsion delivery systems modified with sialic acid-cholesterol. Morphology, particle size, zeta potential, colloidal stability, in vitro hemolysis, biodistribution, and both in vivo and in vitro antitumor efficacy were investigated. The nanoemulsions of vitamin K2 were prepared by a high-pressure homogenizer. The mean particle diameters of the produced nanoemulsions were measured by dynamic light scattering particle analyzer technique after the samples were diluted with distilled water. The nanoemulsion samples were also determined. The average particle sizes of nanoemulsions prepared with a) vitamin K2 and b) sialic acid vitamin K2 nanoemulsion were 119.3 ± 1.3 and 114.8 ± 2.7 nm, respectively. The polydispersity index of both formulations used for nanoemulsion preparation was approximately 0.2, which indicates a narrow particle distribution. The designed vitamin K2 nanoemulsions modified with sialic acid cholesterol conjugate showed good colloidal stability and high antitumor activity [91].

Hasselt and colleagues studied the impact of bile acids on vitamin K oral bioavailability, which was encapsulated inside polymeric micelles. The research showed positive effects of encapsulation in nanoemulsions on the increase in vitamin K absorption inside gastrointestinal tract [104].

### 3.4. Vitamin D

Vitamin D is a precursor of a hormone and has two forms: the first, ergocalciferol (D2) is present in fish and plants, while the second cholecalciferol (D3), is synthesized in skin when exposed to the sun.

Studies published on nanoemulsion for the encapsulation of vitamin D are all quite recent. They have the objective to obtain a fortified system to be used as supplement for all health interventions, which are designed for micronutrient delivery. One of the first studies was published in 2015 by Guttoff et al. They produced vitamin D nanoemulsions by the method of spontaneous emulsification. The organic phase was prepared with vitamin D, medium chain fatty acids (MCT), Tween 20, 40, 60, 80 and 85 as surfactant. Aqueous phase at pH 3 consisted of 0.8% citric acid and 0.08% sodium benzoate. In the next step, the organic phase was titrated into the aqueous phase at a fixed speed using a magnetic stir bar. The results also showed that with the spontaneous emulsification method was able to obtain stable system (particle size lower than 200 nm) with droplet growth lower than 10% in diameter after 1 month of storage [68].

Recently, another method has been also tested to produce vitamin D enriched nanoemulsions. The research was published by Maurya and Aggarwal (2019) who investigated the application of a nanoemulsion fabrication process by phase inversion method in order to encapsulate vitamin D3 for food formulation. The fabrication method had two main two steps: (i) formation of water in oil emulsion by mixing the ingredients (caprylic-/capric triglyceride (CCTG), Leciva S70, vitamin D3, Kolliphor^®^ HS 15, NaCl, water) by using a continuous water bath while shaking. The mixture from ambient temperature was heated to 85°C and then cooled down to 65°C. (ii) Titration of the achieved mixture which is clear brown against hot water at 65°C. The titration was done with continuous stirring which leads to irreversible shock and therefore results in microemulsion breakage system and production of a stable nanoemulsion. In this study five temperature cycles were used in order to cross the zone of phase inversion and achieve a continuous oil phase. Different concentrations of surfactant and oil phase were studied. The nanoemulsion produced with 30% (*v*/*v*) Kolliphor, 20% (*v*/*v*) CCTG and 50 A% (*v*/*v*) water phase was the most suitable in regard of factors such as: emulsion stability, encapsulation efficiency, zeta potential and also showed a high bioavailability of vitamin D. The authors also reported that vitamin D3 release from the nanoemulsion remained sustainable during a significant time period, which indicates high bioavailability of the encapsulated vitamin D3 [54].

Some studies published on vitamin D investigated the production of vitamin D nanoemulsions as carrier, which can be applied for real food products fortification. Stratulat et al. (2015) encapsulated vitamin D3 in two emulsion formulation of flaxseed oil. Calcium caseinate was used for emulsion stabilization in both the presence and absence of lecithin emulsifier. It was also used to standardize the cheese milk and to fortify the cheese in omega-3. The cheese was then stored up to 90 days at 4 °C. The results indicated that the vitamin D3 encapsulation in the cheese and in the form of oil emulsified particles containing lecithin can enhance retention and stability of the vitamin in the curd. Moreover, cheese fortification with vitamin D3 and PUFA had a good influence on the cheese composition, yield, and chemical stability [69].

Recently, Golfomitsou et al. (2018) studied oil-in-water edible nanoemulsions as carrier of vitamin D in order to fortify dairy emulsions. The emulsifiers used for the nanoemulsions were polysorbate 20 and soybean lecithin, while the oil phase consisted of soybean oil or a mixture of soybean oil and cocoa butter. The nanoemulsions were prepared by using a high-pressure homogenizer to obtain at the end a product containing oil droplets with mean particle diameter less than 200 nm. Such nanoemulsions (20%, *w*/*w* of vitamin D) were then applied to fortify a whole fat milk to obtain a product enriched with vitamin D (0.05 μg/mL). The results showed that the droplet diameter of the milk emulsion was not influenced by the presence of the loaded nanoemulsion. Moreover, the milk which was fortified was stable in regard of particle size and gravitational separation for minimum 10 days. Finally, the vitamin which was reported to have radical scavenging activity assessed by electron paramagnetic resonance (EPR) [71].

Besides the application of vitamin D nanoemulsions for food products fortification, interesting research studies have been recently published by Schoener et al., 2019 in order to study the influence of the carriers on vitamin bioaccessibility [74]. or nanoemulsions anti-cancer potential activity in human cells in a study done by Meghani et al., 2018. In detail, Meghani et al. produced O/W nanoemulsions using Tween 80, cinnamon oil and water. The nanoemulsions were fabricated by wash out method, followed by ultrasonication process (20 kHz, 10 min at 400 W with an ultrasonic homogenizer). After obtaining the vitamin D enriched nanoemulsions, cytotoxic and genotoxic assays were performed. Overall, the study highlighted the cytotoxic, genotoxic and antibacterial activities of nanoemulsions enriched with vitamin D loaded in cinnamon oil [72].

Schoener et al. (2019) formulated vitamin D nanoemulsions using pea protein, because of increasing interest in order to substitute both artificial and animal-based food ingredients with plant-based alternatives. Corn, fish, or flaxseed oil were used as carrier oil. Their influence was investigated on colloidal stability, production, and simulated gastrointestinal behavior of nanoemulsions fortified by vitamins. In vitro gastrointestinal study revealed that most of three lipids which were used digested in around the first few minutes in the simulated small intestine. For different oil carriers, bioaccessibility of vitamin was ranked as follows: corn oil > flaxseed oil ≈ fish oil. The results of this study suggested that for delivering and encapsulating of vitamin D3 monounsaturated rich oils (such as corn oil) are better than polyunsaturated rich oils (such as flaxseed or fish oil) [73].

### 3.5. Carotenoids

Carotenoids are another important group of liposoluble antioxidants, which act as filters for blue light in a human eye. Carotenoids are also natural precursors of vitamin A and its metabolites and play an important role in the immune system and formation of cells and tissues [105]. Carotenoids are contained in many vegetables and fruits (e.g., tomatoes, peppers and carrots) and are responsible for their orange, yellow and red color. Carotenoids can be categorized into two main groups: carotenes—α-carotene, β-carotene, γ-carotene, and lycopene; and, xanthophylls—lutein, zeaxanthin, α-cryptoxanthin, and β-cryptoxanthin [36]. Most carotenoids can be found in vegetables and fruits, but they can be in some microbial and edible animal products. Carotenoids have been proved to have a wide a range of positive biological activities [106]. The antioxidant property of carotenoids is due to its binding ability with a singlet oxygen by conjugated double bonds systems [107]. Despite of many health benefits potential of carotenoids, their chemical instability and low water solubility limits their application in many functional beverage and food products [55]. Thus, carotenoids encapsulation by nanoemulsions has been reported to overcome the solubility problems and increase their bioavailability.

Several studies describe encapsulation of carotenoids using nanoemulsions. In the study of Ha and colleagues, nanoemulsions of lycopene were prepared in order to preserve the antioxidant activity and enhance tomato extract bioaccessibility [58]. The nanoemulsion was already enriched with lycopene (contained 6% of lycopene) by the method of emulsification evaporation. For this, tomato extract enriched with lycopene was dissolved in ethyl acetate while stirring for 3 h at 500 rpm. The organic solution and aqueous solution containing 0.5% (*w*/*w*) Tween 20 in distilled water were mixed with each other at constant stirring. The mixture was then homogenized at 5000 rpm for 5 min by a shear homogenizer, and accordingly by the high-pressure homogenizer for 1, 2, and 3 cycles at various pressures of 60, 80, 100, and 140 MPa. Under homogenization pressure in between 60 and 140 MPa (3 cycles), the mean droplet diameter of the resulting nanoemulsion was between 96 and 282 nm. The results showed that the lycopene encapsulated in nanoemulsions with droplet size less than 100 nm had the highest in vitro bioaccessibility.

In another recent study, astaxanthin and lycopene were encapsulated in O/W nanoemulsions by high pressure homogenization using Tween 20 surfactant and linseed oil as oil phase. Droplets mean diameter was studied at different pressures of homogenizer (5, 10, 15, 30, 70 and 100 MPa) and different cycles numbers (1–10). The average particle size of control emulsion sample was obtained at 210, 168 and 164 nm, for 1, 4 and 10 cycles, respectively, at 70 MPa and 242, 134 and 126 nm at the pressure of 100 MPa. Zeta potential of emulsions ranged between −30 and −45 mV, suggesting electrostatic stability. The authors demonstrated that the stability of nanoemulsion during storage was enhanced by addition of an antioxidants. In particular, 6-Hydroxy-2,5,7,8-tetramethylchromane-2-carboxylic acid (Trolox) showed a synergistical reaction with BHT [79].

The effect of the lipid phase on lycopene loaded emulsions has been recently investigated. Oil-in-water beverage emulsions were used for lycopene encapsulation. The oil phase (3% *w*/*w*) was prepared with different ratios of long and short chain triglycerides (LCT to SCT) as follows (*w*/*w*): 100:0, 75:25, 50:50, 25:75, and 0:100 (corn oil:tributyrin). Coarse emulsion prepared by using an ultra-compact digital mixer system for 5 min at 1000 rpm. Nanoemulsions were prepared by passing a coarse emulsion through a single-stage valve microfluidizer at 50 MPa for two cycles. The samples prepared using a low LCT to SCT ratio (0:100) physically were not stable mainly because of Ostwald ripening phenomena. Besides, the rate of creaming in emulsion samples prepared with high LCT was higher, whereas their transparency compared to the beverages with high SCT-to-LCT ratios was lower. The oil particle size significantly decreased for emulsions prepared with corn oil (2.6 μm) in comparison to tributyrin (5.4 μm). The results indicated with addition of LCT the bioaccessibility of lycopene was significantly improved [80].

**Figure 6:**
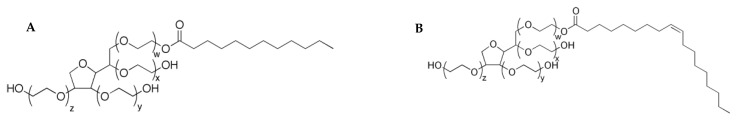
Chemical structure of Tween 20 (**A**) and Tween 80 (**B**).

In another study, lutein was encapsulated in nanoemulsions using medium chain triglycerides (MCT). The nanoemulsions were prepared using spontaneous emulsification approach with sodium benzoate and non-ionic surfactants including Tween 20, 40, 60, 80, and 85. Tween 80 was proven to be the most effective emulsifier. The best results were obtained in systems for which their final formulation consist of 10 wt% oil phase (0.12 wt% lutein +9.88 wt% medium chain triglycerides) and Tween 80 in 10 wt%, and aqueous phase in 80 wt%. The prepared nanoemulsions were stable in regard to both droplet aggregation and gravitational separation while kept at ambient temperature for a shelf life of 1 month. The authors observed that emulsions prepared using Tween 80 provided the smallest droplet size with the narrowest distribution. On the other side, Tween 20 provided emulsions with the largest droplets with the broadest distributions. This system underwent rapid phase separation, with the oil droplets moving rapidly to the top inducing a rapid creaming due to the relatively large droplet size. An explanation to the different behavior of the two surfactants can be found in their hydrophilic–lipophilic balance value. Tween 20 has an appreciably higher hydrophilic–lipophilic balance value compared with the other types of surfactants, which means that it is more hydrophilic with a high tendency of moving into water and a lower solubility in the oil phase. Moreover, they present differences in their chemical structure (as shown in Figure 6) influencing the interfacial curvature, flexibility and the type of structures formed by surfactant–oil–water mixtures [81].

### 3.6. β-Carotene

Studies related to the encapsulation of β-carotene in aqueous based formulations are limited due to their low water solubility. In 2012, Qian and colleagues studied the effect of the antioxidants on chemical degradation of β-carotene encapsulated in nanoemulsions. The compound was incorporated into an O/W nanoemulsions stabilized by a globular protein (β-lactoglobulin) or Tween 20 as a non-ionic surfactant. A strong chelating agent, such as ethylenediaminetetraacetic acid (EDTA), water-soluble antioxidants such as ascorbic acid or an oil-soluble antioxidant (vitamin E acetate)were added to this formulation along with coenzyme Q10. Nanoemulsions were then kept at neutral pH and both their physical and chemical stability were investigated at 55°C. The degradation of β-carotene was monitored by a nondestructive method which was color reflectance measurements. The results showed that oil in water nanoemulsions were prone to color fading in about 3 days of storage time. The degradation was related to the chemical degradation of the carotenoid. The addition of water-soluble antioxidants effectively retarded the degradation. In detail, EDTA had a high influence on inhibition of the color loss. This effect was due to its ability to strongly chelate and inactivate the metals such as iron transition which normally can promote the oxidation of carotenoid. Ascorbic acid could slow the color fading, but it was found to be less effective than EDTA. In between oil soluble antioxidants, coenzyme Q10 could bring a higher protection against color loss in comparison to vitamin E. One reason for this can be its ability to reproduce other antioxidants which presented in the system [82].

In another study, the same research group investigated the effect of temperature, ionic strength, pH and emulsifier on the physical and chemical stability of nanoemulsions enriched with β-carotene. The pH in the rage between 3 and 8 was prepared with different amounts of NaCl (0–500 mM) added in order to have same droplet concentrations. However, the nanoemulsion was unstable at pH lower than 4 due to the precipitation of the protein close to its isoelectric point and at ionic strength higher than 200 mM of NaCl. To test the effect of the temperature, the nanoemulsions were kept in a dark place at 5, 20, 37 and 55 °C for the duration of 15 days. The results showed that the rate of β-carotene degradation was higher for nanoemulsions stored at higher temperatures. On the other side, two different types of emulsifier were tested: β-lactoglobulin and Tween 20. Among the types of emulsifier, β-lactoglobulin was able to induce a higher protection of the bioactive compared to Tween 20 [55].

In the same year, Liu et al. (2012) investigated the impact of the interfacial structure on the physical characteristics, microstructure changes and bioaccessibility of high-pressure homogenized β-carotene nanoemulsions in analysis of in vitro digestion. The model used for digestion by the authors involved the preparation of solutions simulating the compositions of the gastric, duodenal and intestine juices. Their results showed that the food matrix composition significantly affected the release of carotenoids and their bioaccessibility. In detail, the type of surfactant used for the nanoemulsions influenced the digestion. Emulsions prepared by decaglycerol monolaurate (ML750) showed a high release rate without changes in both particles size and discrepancy as well as electrical charge of particles in the gastric fluids. In contrast, whey protein isolate-stabilized emulsions reported a drastic droplet changes with an important modification of their microstructure at the in vitro duodenal stage. Overall, the study was able to discover a β-carotene release which is target-dependent in intestine of these emulsifiers. However, the number of studies is still limited in order to better investigate the effect of other types of emulsifiers considering also in vivo studies [84].

In a more recent study, Sharif et al. investigated the stability of α-tocopherol and β-carotene enriched nanoemulsions. In detail, β-carotene was co-encapsulated with α-tocopherol by adding flaxseed oil or medium chain triglycerides (MCT) as carrier oils and Tween-80 or octenyl succinic anhydride modified starch or as emulsifiers. The produced nanoemulsions were kept for a shelf-life of 4 weeks and the impact of the temperature on their oxidative stability was studied by measuring peroxide value and thiobarbituric acid reactive substances. The results showed that adding α-tocopherol for the protection of β-carotene in MCT-based emulsions worked as an antioxidant. However, it could not protect β-carotene degradation in flaxseed oil-based emulsions. When an antioxidant such as eugenol (1%, *w*/*w*) was added to the product, the oxidative stability enhanced and the retention of both β-carotene and α-tocopherol increased in all emulsions. Nanoemulsions which was contained β-carotene and α-tocopherol and eugenol and stabilized by octenyl succinic anhydride modified starch showed a retention of β-carotene equal to 42% and α-tocopherol equal to 90% during 4 weeks of storage at 40°C. This research represented an important starting point for the simultaneous encapsulation of lipophilic bioactives as well as development of functional foods and beverages [108].

All the studies mentioned so far on β-carotene-enriched nanoemulsions applied high energy intensive methods applying an intensive mechanical mixing of the coarse emulsions followed by a homogenization at high pressures. The only study testing a different method was tested by Silva et al. (2011) who prepared β-carotene-enriched nanoemulsions replacing the microfluidizer with a conventional homogenization. The main objectives of the research were: (1) to prepare nanoemulsions by applying high-energy emulsification method and to study the impact of different processing factors on the stability of the nanostructures, (2) to determine the physical characteristics of the obtained nanoemulsions. The O/W emulsions were prepared dissolving β-carotene (0.03%, *w*/*w*) in n-hexane at 40 °C. The organic solution was then added to an aqueous solution, which contains 0.5% *w*/*w* of Tween 20. The coarse emulsions were prepared by using an Ultra-Turrax with the mixing speed ranging from 3500 to 6500 rpm (min-1, number of cycles from 1 to 3 and mixing times from 2 to 8 min. At these processing conditions, the volume-surface diameter of nanoemulsions was ranged from 9 to 280 nm and showed a monomodal size distribution. The shelf-life study which was performed for 21 days at 4 °C in dark, showed a good physical stability of the systems, although the degradation of β-carotene occurred over time. Overall, the study provided an alternative for producing β-carotene nanoemulsions with an inexpensive technique. However, the use of n-hexane as solvent should be carefully reviewed due to its toxicity if small residues remained in the food formulation [45].

### 3.7. Polyunsaturated Fatty Acids (PUFA)

Polyunsaturated fatty acids (PUFA) are responsible for reducing risk of chronic diseases, such as cardiovascular disease, inflammation, immune response disorders, mental disorders, and poor infant development can be mentioned [75]. The most important PUFA in human diet are linoleic, alpha-linolenic acids, eicosapentaenoic acid (EPA), and docosahexaenoic acid (DHA). Thus, there is an increased interest in encapsulation of PUFA for food fortification [109].

The study of Lane et al. in yoghurt demonstrated that nanoemulsion delivery systems may enhance omega-3 fatty acids absorption [110]. Soy lecithin in combination with ultrasound emulsification method was used to create a nanoemulsion, which contains 50% DHA algae oil and water. The resulting nanoemulsion with droplets size of 258 nm afterwards was added to strawberry yoghurt. The results showed increased bioavailability of the PUFA after application of nanoemulsion technology in comparison to a bulk oil-enriched yogurt. In addition, the work of Dey et al. showed that EPA-DHA rich fish oil bioavailability was higher in nanoemulsion. In this work nanoemulsions were prepared by a microfluidizer process carrying out 10 passes at 40,000 psi. Tween 20 and Span 80 were used in a ratio of 1:1 (*w*/*w*) and the ratio of oil:surfactant:water was maintained at 1.5:1:100 (*w*/*w*/*w*) respectively. The authors showed that also the polyethylene ester based surfactants (i.e., Span 80 and Tween 20) facilitated mucosal transport of the emulsified lipids [63].

In the recent study of Karthik et al. DHA algae oil water nanoemulsions were prepared by high pressure homogenizer with the addition of different surfactants: Tween-40, sodium caseinate and soy lecithin. Nanoemulsion prepared by Tween 40 had lower particle size (148 nm) compared to sodium caseinate (206 nm) and soy lecithin (760 nm), respectively. Structural properties of nanoemulsion prepared by Tween 40 were retained during shelf-life, while nanoemulsions prepared by adding sodium caseinate showed flocculation and coalescence phenomena. The fatty acids profile of the nanoemulsions did not change during storage time. Moreover, lower peroxidation was obtained by nanoemulsions prepared by Tween 40 as well as a higher stability and lipid digestibility extent [62].

In another work of Lane et al., O/W nanoemulsions were prepared using vegetarian PUFA omega 3 oils (flaxseed and algae) and ultrasound methods. Tween 40 and lecithin were used as surfactants in nanoemulsion preparation. The obtained mean particle size of nanoemulsions was 192 nm for flaxseed and 182 nm for algae oil. For nanoemulsion preparation, first a rotor stator mixer at 4000 rpm for 2 min was used, followed by a secondary homogenization in ultrasound sonicator at 24 kHz. Nanoemulsions with up to 50% (*w*/*w*) of oil were produced. Droplet sizes were affected by the ratio of oil:water and increased significantly with higher loads of oil. The results proved that producing nanoemulsions with 70% (*w*/*w*) oil load was not possible since phase inversion happened at this level. Statistical analysis indicated that the duration of ultrasound also had significant impact on nanoemulsion droplet sizes. A processing time between 10 and 12 min was found to be optimum in order to create the smallest droplets size [78].

In the work of Costa and colleagues, nanoemulsions were prepared using an oil extracted from microalga Spirulina sp. LEB18. Biopeptides obtained from Spirulina sp. LEB18 were also added during nanoemulsion preparation. The aqueous phase was prepared by dissolving Tween 80 in distilled water. The microalgal lipid oil phase was produced by a mixture of chloroform-methanol (2:1) solvent and biopeptides. In this study, the nanoemulsion mean particle size was measured in the range 222.9 ± 3.4 to 466.9 ± 5.3 nm. The nanoemulsions were prepared by simultaneously mixing with Ultra-Turrax at the speed of 10,000 rpm in an ultrasonic bath at frequency of 50/60 Hz. Due to the results of the polydispersity index and zeta potential the nanoemulsions were stable during a shelf life of 14 days [83].

In the study of Gulotta et al. fish oil rich in PUFA was encapsulated by using a spontaneous emulsification. A mixture of fish oil (from 0% to 10 wt %), lemon oil (0−10 wt %) medium chain triglycerides, and Tween 80 as a nonionic surfactant (2.5−10 wt %) was used. The water phase was prepared by a buffer solution at pH 3.0, which consisted of 0.8% citric acid and 0.08% of sodium benzoate mixed with different percentages of propylene glycol, glycerol, or ethanol (0−50 wt %). The authors investigated the impact of the surfactant and the oil ratio on the mean particle diameter. A mean diameter smaller than 200 nm was achieved with surfactant-to-oil ratio greater than 0.75. Moreover, the impact of the cosolvent type (ethanol, propylene glycol, and glycerol) and the concentration on the droplet particle diameter was measured. The results indicated that the smallest droplets size (51 ± 4 nm) was achieved in nanoemulsion formulation containing 40% glycerol [74].

Another study by Belhaj et al. describes salmon oil encapsulation by high-pressure homogenization. The homogenization process was repeated for five times at 22,000 psi (1700 bar) under the flow of nitrogen. Marine lecithin, a mixture of phospholipids extracted from salmon heads by Folch method (1%) was used as surfactant. Nanoemulsions with particle size of 160–207 nm were obtained. The results indicated that addition of natural antioxidants, such as tocopherols and astaxanthin, provided a better encapsulation protection for crude salmon oil. The addition of 0.2% α-tocopherol showed a pro-oxidant effect during storage at 30°C, while the addition of quercetin slightly enhanced the oxidative stability of oils in nanoemulsion [75].

Recently, O/W nanoemulsions were prepared by high pressure homogenization using high oleic palm oil (1–20% *w*/*w*) in combination with (1–20% *w*/*w*) whey protein, Tween 20 (1:1 *w*/*w* ratio) and water as an aqueous phase. In this study, by applying two cycles in the microfluidizer at 20,000 psi, the authors could achieve the minimum particle size avoiding the effect of coalescence. Moreover, high oleic palm oil in low concentration (1% *w*/*w*) and whey protein (1% *w*/*w*) could produce stable nanoemulsions. Values of zeta potential varied between 29.7 mV and −47.2 mV. The results indicated that storage of nanoemulsions at 19 °C for 4 days had an impact on the viscosity. The viscosity increased around six-fold compared to the fresh sample [77].

### 3.8. Essential Oils and Flavor Compounds

Essential oils are botanical products, which principally derive from whole or specific parts of plants which includes flowers, roots, barks, leaves, seeds, peel, fruits and wood. Apart from their discovered aromatic and coloring properties, essential oils found to have antimicrobial and antioxidant activity [111]. The hydrophobic, volatile and reactive nature of essential oils reduces the possibility of their incorporation directly into food matrices. Encapsulation in nanoemulsions can help to overcome the challenge of essential oils incorporation into food formulations. Common techniques for production of nanoemulsions with essential oils include two methods [112].

One method is essential oil homogenization in the aqueous solutions in presence of emulsifier. For example, in a recent study, a mixture of (a) 4% *w/v* sodium caseinate and 0.5% *w/v* lecithin or (b) 2% *w/v* sodium caseinate and 0.25% *w/v* lecithin was used in order to emulsify thyme oil by high pressure homogenization [90]. The average droplet size of 82.5 nm for the first combination and 125.5 nm for the second was obtained. In another work nanoemulsions of eugenol, the main bioactive of many essential oils, were prepared with lauric arginate and lecithin, resulting in spherical droplets lower than 100 nm [91]. In both cases antimicrobial and antioxidant properties of nanoemulsions were similar to those of essential oils. According to Sharma et al., also clove and lemongrass oil antifungal activity was improved by incorporation into O/W nanoemulsions [64].

The other method was based on essential oil pre-dissolution in typically used oils, followed by emulsification in the aqueous phase. In the study of Yang et al., nanoemulsions were produced by the combination of citrus oils: sweet orange oil and bergamot oil. Common triacylglycerol oils were used as oil phase, for instance: corn oil and medium chain triglyceride oil with variable mixing ratios. The results indicated that nanoemulsions stability which consist of a mixed oil was significantly higher in comparison to nanoemulsions prepared by pure citrus oils [97].

Many published studies have also showed a positive effect of mixing essential oils with other lipids on physical stability of nanoemulsions and their droplet size. For example, in the work by Liang et al. peppermint oil was encapsulated by nanoemulsions technique in pure state and mixed with medium chain triglyceride using maize starch as supernatant. Pure peppermint oil, MCT, and their mixture at 1:5, 1:1, and 5:1 (*v*/*v*) ratio as oil phase were mixed with aqueous phase were premixed with Ultra-Turrax at 24,000 rpm for 1 min at the room temperature. It was then passed through a high-pressure homogenizer at 50, 100, and 150 MPa for 1, 3, 5, 7, 10, 15, or 20 cycles respectively. The results showed that addition of MCT brought the droplet size from microscale (4 µm) to nanoscale. Moreover, the mean particle size continuously decreased from first to the last cycle. An increase in pressure from 50 to 100 MPa could result in a significant decrease at particle size. However, application of higher pressures from 100 to 150 MPa in nanoemulsion preparation did not make a significant change in droplet size. The resulting nanoemulsions remained stable for 30 days at 25 °C. Additionally, the study on thyme oil the addition of corn oil (50–75%) resulted in positive effect on particle size and physical stability of nanoemulsions [113].

However, in some cases nanoemulsions with pure essential oils were successfully prepared. For instance, the lemongrass essential oil with Tween 80 and sodium alginate as surfactant [95] and oregano, thyme, lemongrass and mandarin essential oil in nanoemulsions stabilized with Tween 80 and high methoxyl pectin [100].

## 4. Conclusions

The present review gives an insight regarding current advances in the technological and practical aspects of encapsulation of liposoluble bioactives by nanoemulsions. There are different structured nanoemulsion methods including low-energy and high-energy methods widely available for the successful encapsulation. Oil-in-water nanoemulsions remain the most widely used encapsulation systems of lipid-soluble bioactives in the food industry. Among lipid-soluble vitamins, most studies have focused on encapsulation of tocopherols, carotenoids and vitamin D by nanoemulsions, while the knowledge on vitamins A and K is still limited. There is increasing interest to use nanoemulsions for encapsulation of polyunsaturated fatty acids and essential oils.

The main challenges are linked to optimization of encapsulation conditions and the choice of the emulsifiers to stabilize nanoemulsions. Core materials, emulsifier type and production conditions have a significant impact on both the chemical and physical stability of nanoemulsions and need to be investigated and optimized. The authors are aiming to study properties of the aqueous phase (polarity and ionic strength), lipid phase (polarity, physical state, composition), and surfactant (small molecular weight vs. biopolymer) in order to obtain stable nanoemulsions. However, extensive chemical and physical characterization of the resulting nanoemulsions is limiting the number of parameters that can be studied in one study. Overall, the current research shows droplets with reduced diameter or radius are more stable to aggregation and creaming, which leads to better encapsulation of lipid-soluble bioactives and more stable nanoemulsions. A better understanding of the factors having impact on the nanoemulsion stability will allow the prevention of bioactive degradation, providing a longer shelf-life and presenting the benefits of nanoemulsion technology in food applications.

According to recently published studies, the chemical stability and bioavailability of lipid-soluble bioactives can be significantly improved by encapsulation in nanoemulsions. The small diameter of the oil droplets increases their surface-to-volume ratio which, in the case of encapsulated bioactive molecules, increases their absorption during digestion. In summary, nanoemulsions are an excellent means for encapsulation of lipid-soluble bioactives in food applications.

## Figures and Tables

**Table 1:** Typical emulsifiers used in food nanoemulsions [41].

Surfactant Type	Surfactant Origin	Examples
Low-Molecular-Weight Surfactants	Synthetic	esters of sucrose mono- and diglycerides derivatives of monoglycerides polyoxyethylene derivatives (Tween series, Span series, sucrose monopalmitate) derivatives of monoglycerides
	Natural	phospholipids (phosphotidyletanolamine, phosphatidylcholine, phosphatidic acid, phosphatidylinositol glycolipids (rhamnolipids, sophorolipids, trehalolipids, cellobiose lipids and mannosylerythritol lipids) saponins (*Quillaja* saponins, tea saponins, ginseng saponins)
High-Molecular-Weight Emulsifiers	Proteins	animal proteins (mainly from milk—whey protein, casein, β-lactoglobulin, sodium caseinate) plant proteins (soy protein isolate, β-conglycinin, glycinin, pea proteins, lentil proteins) mixed proteins (e.g., sodium caseinate + micellar casein)
	Polysaccharides	gum arabic, corn fiber gum pectin (high methoxylated pectin, ultra-high methoxylated pectin) plant mucilage (from the leaves of pereskia aculeata miller, yellow mustard mucilage) Octenyl succinic anhydride (OSA)-modified polysaccharides (OSA-modified starch, OSA-β-cyclodextrin, OSA-Konjac Glucomannan)

**Table 2:** Application of nanoemulsions for encapsulation of lipid-soluble bioactives.

Bioactive Compound	Nanoemulsion Production	Type of Emulsion	Surfactant, Emulsifier, Oil Phase	Particle Size	Reference
Vitamin D	Spontaneous emulsification	Oil-in-water	Tween 20, 40, 60, 80, 85 Oil phase: MCT ^1^	<200 nm	[68]
Vitamin D	Ultra-Turrax: 17,000 rpm, 2 min. HPH ^2^: 70 MPa for 2 cycles. 35 MPa for 1 cycle	Oil-in-water	Calcium caseinate (3%, *w*/*v*) and soy lecithin 2%	- ^8^	[69]
Vitamin D_3_	High speed blender HPH ^2^: 83 MPa, for 3 cycles.	Oil-in-water	Active saponins 2% Oil phase: fish oil, corn oil, MCT ^1^, mineral oil, and orange oil	0.14–0.19 μm	[70]
Vitamin D_3_	Magnetic stirrer HPH ^2^: 10 MPa, 10–15 passages	Oil-in-water	Tween 20 Oil phase: soybean oil, lecithin, cocoa butter	174 ± 7 nm; 26 ± 3 nm.	[71]
Vitamin D	Ultrasonic homogenizer: 20 kHz,10 min, 400 W	Oil-in-water	Tween 80 Oil phase: cinnamon oil Water phase: PBS ^3^, water, DMEM ^4^ F12 media	166.2, 118.0, 170.8 and 40.52 nm	[72]
Vitamin D_3_	Phase inversion	Water-in-oil	Soybean derived lecithin Oil phase: Monegyl Caprylic-/capric triglyceride	39.12 ± 0.33–64.11 ± 1.93 nm	[54]
Vitamin D_3_	High-speed blender HPH ^2^: 83 MPa, for 5 cycles	Oil-in-water	Pea protein Oil phase: flaxseed oil, corn oil, or fish oil	0.34 μm	[73]
PUFA ^5^	Spontaneous emulsification	Oil-in-water	Tween 80 (2.5–10 wt %). Oil phase: MCT ^1^, lemon oil.	>1000 nm at low surfactant levels, <200 nm at high surfactant levels	[74]
Salmon oil	HPH ^2^: 17 MPa, 5 cycles	Oil-in-water	Marine lecithin (mixture of phospholipids)	160–207 nm	[75]
DHA ^6^ algae oil	Ultrasound emulsification	Oil-in-water	Soy lecithin	258 nm	[76]
DHA algae oil	Ultra-Turrax: 1000 rpm for 10 min HPH ^2^: 90 MPa, 5 and 7 cycles; 100 MPa, 5 and 7 cycles	Oil-in-water	Tween 40, Sodium caseinate Soya lecithin	148 nm; 206 nm; 760 nm	[62]
High-oleic palm oil (1–20% *w*/*w*)	Ultra-Turrax: 9500 rpm HPH ^2^: 68–138 MPa, for 1, 2 and 3 cycles	Oil-in-water	Whey (1–20% *w*/*w*)	163.7–2268.0 nm	[77]
Flaxseed/high DHA ^4^ algae oil	Ultra-Turrax: 4000 rpm for 2 min, Ultrasound sonication: 24 kHz	Oil-in-water	Soy lecithin, Tween 40	192 nm; 182 nm	[78]
Fish oil	HPH ^2^: 275 MPa, for 10 cycles	Oil-in-water	Tween 20, Span 80, ratio 1:1 (*w*/*w*) Oil/surfactant/water ratio 1.5:1:100 (*w*/*w*/*w*)	89.7 ± 27.7 nm	[63]
Lycopene-enriched tomato extract	Ultra-Turrax: 5000 rpm for 5 min HPH ^2^: at 60, 80, 100, and 140 MPa for 1,2 and 3 cycles	Oil-in-water	Tween 20	96 ± 12 nm	[58]
Astaxanthin and lycopene	Ultra-Turrax: 5000 rpm for 10 min HPH ^2^: 5, 10, 15, 30, 70 and 100 MPa for (1–10) cycles.	Oil-in-water	Tween 20, 0.5% *w*/*w* Oil phase: linseed oil (1% *w*/*w*)	1, 4 and 10 cycles at 70 MPa (210, 168, 164 nm); 100 MPa: 242, 134, 126 nm.	[79]
Lycopene	Ultra-mixer: for 5 min at 1000 rpm, HPH ^2^: 50 MPa for 2 cycles	Oil-in-water	WPI ^7^ (3%) Oil phase: short or long chain triglycerides (tributyrin and corn oil).	2.6 μm; 5.4 μm	[80]
Lutein	Spontaneous emulsification	Oil-in-water	Tween 20, 40, 60, 80, 85, sodium benzoate Oil phase: corn oil or MCT	190–270 nm with MCT 192 nm no lutein, 205 nm with 0.2% lutein, 270 nm with 1.2% lutein	[81]
β-carotene	Ultra-Turrax homogenizer HPH ^2^: 62 MPa, for 3 cycles	Oil-in-water	β-lactoglobulin Oil phase: orange oil	78 nm	[82]
β-carotene	Ultra-Turrax homogenizer	Oil-in-water	Tween 20 (0.5%)	9.24 ± 0.16–276.77 ± 17.70 nm	[45]
Spirulina oil	Ultra Turrax: at 10,000 rpm Ultrasonic bath at 50–60 kHz	Oil-in-water	Spirulina peptides Tween 80 (0, 0.5 and 1% *v*/*v*)	222.9 ± 3.4–466.9 ± 5.3 nm	[83]
β-carotene	Ultra Turrax HPH ^2^: 60 MPa for 3 cycles.	Oil-in-water	Whey protein isolate (4%), soybean soluble polysaccharides (4%), decaglycerol monolaurate (4%) Oil phase: MCT	579.45–1829.50 nm	[84]
β-carotene	Ultra Turrax HPH ^2^: 62 MPa, for 3 cycles	Oil-in-water	β-lactoglobulin (2%) Tween 20 (1.5%) Oil phase: corn oil	<500 nm	[82]
β-carotene	Ultra Turrax: 18,000 rpm, 3 min HPH^2^: 100 MPa for 5 cycles	Oil-in-water	Tween 80 (2%) OSA-starch (2%) Oil phase: flaxseed oil and MCT	123.9–185.6 mn	[38]
β-carotene	Ultra-Turrax: 14,500 rpm, 2 min, HPH ^2^: 69 MPa, for 6 cycles	Oil-in-water	Tween 20 (1%) Oil phase: 70% corn oil, 30% Span 80 and β-carotene (0.02%)	300 nm	[57]
Vitamin A	HPH ^2^: 150 MPa for 8 cycles	Oil-in-water	Egg phosphatidylcholine Oil Phase: corn oil (4.5–15%)	<236.8 ± 26.9 nm	[85]
Vitamin A	Emulsification method with two steps	Oil-in-water, Water-in-oil, Oil-in-water-in-oil	1,3-butanediol Emalex 600 di-IS Oil phase: Liquid paraffin	-	[60]
Vitamin A	Ultra-Turrax: 7000 rpm, 10 min	Oil-in-water	Tween 80 Oil Phase: Poly (methyl methacrylate-*co*-methacrylic acid	-	[86]
Vitamin A	Ultra-Turrax: 24,000 rpm, 4 min HPH ^2^: 7 MPa, for 3 cycles	Oil-in-water	Lecithin (10%) Ethanol (10%)	475.7 nm	[87]
Vitamin A	Ultra-Turrax: 5000 rpm, 10 min HPH ^2^: 200 Mpa	Oil-in-water	Whey protein isolate (4.3) Oil phase: peanut oil (30%)	<300 nm	[88]
Vitamin A in palmitate/peanut oil	HPH ^2^: 172 MPa, for 7 cycles	Oil-in-water	Lecithin/Saponin (1%)	115 nm	[89]
Vitamin A	Ultra-Turrax: 720, 846.7 and 955.8 rpm.	Oil-in-water	Whey protein isolate Virgin coconut oil (50%)	5–20 μm	[90]
Vitamin K1	A syringe pump was used to add organic phase to oil phase (flow rate 50 mL/min)	Oil-in-water	Tween 80 Oil phase: 1,2-dioleoyl-sn-glycero-3-phosphoethanolamine-N-	<253.9 nm	[61]
Vitamin K	HPH ^2^: 14,000 psi, 4 cycles	Oil-in-water	Lipoid E80 (1.5%) Oil Phase: MCT ^1^ (10%)	<119.3 ± 1.3 nm	[91]
Vitamin E	Ultra-Turrax: 2 min HPH^2^	Oil-in-water	Whey protein isolate (3.5%) Oil phase: Orange oil (3.5%)	-	[92]
Vitamin E	Ultra-Turrax HPH ^2^: 83 MPa, 3 cycles	Oil-in-water	Gum arabic-Quillaja saponin-whey protein isolate (1.5%) Oil phase: corn oil (80%)	<100 µM	[59]
Vitamin E	Ultra-Turrax: 500 rpm, 25 °C	Oil-in-water	Tween 20-40-60-80-85 (10%)	<50 nm	[93]
Vitamin E	Ultra-Turrax: 10,000 rpm for 10 min. HPH ^2^: 20–50 MPa, 3 cycles.	Oil-in-water	Tween 40 Oil phase: Palm Oil (10%)	<1000 nm	[94]
Vitamin E	Ultra-Turrax: 2 min at room temperature HPH ^2^: 83 MPa, 3 cycles	Oil-in-water	Whey protein isolate and gum arabic Oil Phase: Orange Oil (5%)	<0.38 µM	[70]
Thyme oil (1% *w*/*v*)	Ultra-Turrax: 15,000 rpm for 3 min	Oil-in-water	Sodium caseinate and soy lecithin	82.5 nm; 125.5 nm	[95]
Thymol and eugenol	Ultra Turrax: 15,000 rpm for 6 min	Oil-in-water	Lauric arginate and soy lecithin	55 (eugenol) and 75 (thymol) nm	[96]
Clove and lemongrass oil	Spontaneous emulsification	Oil-in-water	Tween 20, Castor Oil Ethoxylate-40,	76.73 nm	[64]
Bergamot oil and sweet orange oil	Ultra Turrax: 9000 rpm for 3 min HPH ^2^: 60 MPa for 3 cycles	Oil-in-water	Tween 80, soy lecithin Oil phase: citrus oil mixtures with corn oil and MCT ^1^ oil	30 nm (5% oil phase); 190 nm (15% oil phase)	[97]
Peppermint oil	Ultra-Turrax: 24,000 rpm for 1 min, HPH ^2^: 50, 100, and 150 MPa for 1, 3, 5, 7, 10, 15, or 20 cycles.	Oil-in-water	Starch Oil phase: peppermint oil, MCT ^1^ (pure oils), and mixtures 1:5, 1:1, and 5:1 (*v*/*v*)	146.0 ± 1.5 nm; <200 nm	[97]
Thyme oil	Ultra-Turrax: 60 s HPH ^2^: 0.01 MPa, 5 cycles	Oil-in-water	Oil phase (5% *w*/*w*): thyme oil, corn oil (from 0 to 100 wt % corn oil) pH 4.0 Tween 80 (0.5 *w*/*w*)	163 nm	[98]
Oregano, thyme, lemongrass and mandarin essential oils (1% *w*/*v*)	Ultra Turrax: 9500 rpm for 2 min HPH ^2^: 150 MPa, 5 cycles.	Oil-in-water	High methoxyl pectin (1% *w*/*v*) and Tween 80 (5% *w*/*v*)	<50 nm	[99]
Lemongrass oil	Ultra-Turrax: 3400 rpm for 2 min. HPH ^2^: 50, 100 or 150 MPa (1, 2, 3, 4, 5 and 10) cycles	Oil-in-water	Sodium alginate (1% *w*/*v*) and Tween 80 (1% *v*/*v*)	53 ± 5 nm, 46 ± 7 nm, 23 ± 2 nm 7.35 ± 1.67 nm	[100]

^1^ MCT: Medium-chain triglycerides; ^2^ HPH: high pressure homogenization; ^3^ PBS: Phosphate-buffered saline; ^4^ DMEM: Dulbecco’s Modified Eagle’s Medium/Nutrient Mixture F-12; ^5^ PUFA: polyunsaturated fatty acids; ^6^ DHA: docosahexaenoic acid; ^7^ WPI: Whey protein isolate, ^8^ -: information not available.
